# Associated factors of diabetic retinopathy in type 1 and 2 diabetes in Limpopo province in South Africa

**DOI:** 10.3389/fcdhc.2024.1319840

**Published:** 2024-05-02

**Authors:** Khisimusi Debree Maluleke, Cairo Bruce Ntimana, Reneilwe Given Mashaba, Kagiso Peace Seakamela, Eric Maimela

**Affiliations:** Dikgale Mamabolo Mothiba (DIMAMO) Population Health Research Centre, University of Limpopo, Polokwane, South Africa

**Keywords:** prevalence, diabetic retinopathy, diabetes mellitus, blindness, non-proliferative

## Abstract

**Background:**

Diabetic retinopathy (DR) is the major cause of vision impairment or blindness in individuals who have diabetes. It has accounted for 2.6% of all cases of blindness, and 1.9% of all cases of vision impairments globally. There is a lack of data on the prevalence of diabetic retinopathy and its associated factors amongst diabetic rural populations. Hence, the current study aimed to determine factors associated with diabetic retinopathy (DR) among diabetes mellitus (DM) patients undergoing diabetic therapy.

**Methods:**

The study was cross-sectional in design and the participants were selected using convenient sampling. STATA version 15 software was used for data analysis. Chi-square was used to compare proportions. Logistic regression was used to determine the relationship between DR and associated risk factors.

**Results:**

The prevalence of DR was 35.3%, of which 32% were mild and 3.4% were moderate non-proliferative DR (NPDR). Females were more unemployed than males (32.1% versus 16.8%, *p=0.0058).* Males were found to drink alcohol (21.8% versus 1.9%, *p<0.001*) and smoke cigarettes (4% versus 0.3%, p=0.0034) more than females. Being aged ≥ 55 years (OR: 2.7, 95% CI: 1.6-4.4), with matric qualification (OR: 0.6; 95% CI: 0.4-1.0); employed (OR: 1.4, 95% CI: 1.2-1.6); having high systolic blood pressure (OR=1.4, 95%CI=1.1-1.7) were the independent determinants of DR.

**Conclusions:**

The prevalence of diabetic retinopathy was 34%. DR was determined by high systolic blood pressure, old age, and employment. Although not statistically significant, gender, hyperglycemic state, poor glycemic control, smoking, and increased body mass index (BMI) were associated with increased risk of developing DR.

## 1 Introduction

Diabetic retinopathy (DR) is the major cause of vision impairment or blindness in individuals who have diabetes (1). It is caused by persistent high blood sugar levels, which destroy the blood vessels or capillaries in the retina, resulting in a blockage or leakage from the retinal capillaries (2). In response to intra-retinal hemorrhage and blockage, the retina promotes the formation of new vessels to compensate for the malfunctioning ones (3). The formation of new vessels and/or the release of blood-borne components from damaged vessels can cause substantial vision loss and scarring (4, 5).

In 2010, roughly 800,000 diabetics were deemed blind, while 3.7 million diabetics were identified as visually impaired due to DR, with DR rising rapidly from 27% in 1990 to 64% in 2012 worldwide (6). Diabetic retinopathy has accounted for 2.6% of all cases of blindness, and 1.9% of all cases of vision impairments globally, which is an increase from 2.1% of blindness and 1.3% of vision impairments (6–8)

Risk factors influencing the rapid development of DR in diabetic people include; age (2); duration of diabetes mellitus (DM) (9), poorly controlled DM (10), and elevated BMI (11). Hypertension (12–16), type of DM (11), pregnancy (17), lipedema (9), nephropathy (18), and smoking (1) are also predisposing factors for DR. Other variables that contribute to the occurrence of DR include socioeconomic challenges such as insufficient access to medical care, inadequate facilities, poor loss gross domestic products (GDPs), and insufficient finance for the healthcare (19–22). Moreover, unhealthy lifestyles, physical inactivity, and bad eating habits are reported to be associated with DR development (23, 24).

The global prevalence of DR was estimated to be between 27% and 28.1% (25, 26). The regional prevalence of DR is 20.6% in Europe, 12.5% in South East Asia, 36.2% in the Western Pacific, and 33.8% in Africa and the Middle East region (26). The continued increase in the prevalence of DM in low-to-middle-income countries has contributed to the increased prevalence of DR (27). The prevalence of DR in South Africa was reported to be at 71% in a study conducted in KwaZulu-Natal (28), however, there is little information on the prevalence of DR and its contributing factors among the rural black population in diabetic patients. Given the ongoing rise in diabetes mellitus, which may eventually result in DR, the current study aimed to determine the prevalence and risk factors of retinopathy (DR) among diabetes mellitus (DM) patients undergoing diabetic therapy in the Maruleng sub-district in Limpopo Province.

## 2 Materials and methods

### 2.1 Study design and setting

This cross-sectional survey was undertaken in all ten community clinics and the District Hospital in the Maruleng sub-district, Mopani District, Limpopo Province, South Africa. The Turfloop Research and Ethics Committee (TREC/28/2020: PG) of the University of Limpopo in South Africa gave ethical approval. The Limpopo Department of Health’s Provincial Health Research and Ethics Committee approved permission for this study to be conducted in the specified public healthcare facilities

### 2.2 Sampling

The study consisted of 416 DM patients aged 18 years or older. Participants were selected using convenient sampling. Participants with gestational diabetes were excluded.

### 2.3 Data collection

A piloted structured questionnaire was used to collect data, the questionnaire consisted of socio-demographics, behavioral (cigarette and alcohol drinking habits), history of DM, and treatment compliance information questions. The questionnaire was validated using the following steps: A draft of the questionnaire and a validation form for the validation procedures were submitted to the researchers who are aspect in validating questionnaires. Each item on the form was given a score based on its relevance (1 = not relevant, 2 = somewhat relevant, 3 = quite relevant, and 4 = extremely important), as well as its clarity (1 = not clear, 2 = somewhat clear, 3 = quite clear, and 4 = highly clear). The level of agreement and content validity index were calculated using these scores, and a mean content validity index of 0.80 or higher was considered acceptable (29). The questionnaire was considered valid after it was able to measure the intended parameters when repeated several times. Furthermore, the questionnaire underwent additional validation through a pilot survey involving the initial 8 participants. Blood pressure (BP), glucose, height, and weight were measured by incumbent nursing personnel at the reception area of every facility during the time of the study.

### 2.4 Fundus examination and diagnostic criteria

Eye care personnel performed a fundus examination using indirect ophthalmoscopy with 20 diopters (D) Volk lens to assess retinal integrity on dilated pupils to obtain a detailed view using 1% of Tropicamide eye drops. The presence of retinal abnormalities such as microaneurysms, hemorrhages, hard exudates, cotton wool spots, retinal venous beading, and other retinal microvascular abnormalities like neovascularization (new blood vessel growth) within one-disc diameter (NVD) or elsewhere on the retina (NVE) was used as a diagnostic criterion. Mild non-proliferative DR was diagnosed by the presence of at least one microaneurysm. Moderate non-proliferative DR was diagnosed by the presence of multiple microaneurysms plus hemorrhages. Severe non-proliferative DR was diagnosed by the presence of multiple microaneurysms, hemorrhage or hard exudates or cotton wool spots or venous beadings. Proliferative DR (PDR) was diagnosed by the presence of visible new blood vessels growing on the disc or elsewhere on the retina, etc., based on the Early Treatment Diabetic Retinopathy Study (ETDRS) (1) criteria for international classification/grading of DR.

### 2.5 Statistical analysis methods

Data analysis was carried out using the STATA version 15 for Windows (STATA Corp LP, College Station, TX, USA). Categorical variables were presented as numbers and percentages, which were reported as proportions at a 95% confidence interval (CI). The strength of association between the dependent variable (DR) and the independent variables (age, gender, educational and employment status, systolic blood pressure, hyperglycemia, smoking, and body mass index) was determined using logistic regression analysis, and the results were reported as odds ratios at 95% confidence intervals.

## 3 Results

### 3.1 Sample and socio-demographic information

A total of 416 DM patients receiving diabetic chronic treatment were enrolled in this study, of which the majority (76%) were females, and only 24% were males. **Table 1** below shows that significantly more females than males were unemployed (32.1% versus 16.8%, *p=0.0058*). Males were significantly more likely to drink alcohol (21.8% versus 1.9%, *p<0.001*) and smoke cigarettes (4% versus 0.3%, *p=0.0034*) as compared to females.

**Table 1:** Socio-demographic profile of participants by sex.

Socio-demographic profile		Males (n=101)	Females (n=315)	P value
		**n (%)**	**n (%)**	
Age	24 - 34	0	8 (2.5)	0.0541
	35 - 44	7 (6.9)	22 (7)	
	45 - 54	14 (13.9)	6 (1.9)	
	55 - 64	27 (26.7)	106 (33.7)	
	65 - 74	44 (43.6)	85 (27)	
	75 - 84	9 (8.9)	30 (9.5)	
	≥85	0	3 (1)	
Educational level	No matric certificate	86 (85.2)	268 (85.1)	0.7341
	Matric certificate	8 (7.9)	26 (8.3)	
	Post matric certificate	8 (7.9)	21 (6.7)	
Employment status	Unemployed	17 (16.8)	101 (32.1)	0.0059
	Employed	13 (12.9)	36 (11.4)	
	Self-employed	2 (2)	5 (1.6)	
	Pensioner	69 (68.3)	173 (56.5)	
Alcohol drinking	No	79 (78.2)	309 (98.1)	<0.0001
	Yes	22 (21.8)	6 (1.9)	
Cigarette smoking	No	97 (96)	314 (99.7)	0.0034
	Yes	4 (4)	1 (0.3)	
Diabetic retinopathy	Yes	34 (23.1)	113 (35.9)	0.721
	No	67 (66.3)	203 (64.1)	
Diabetes	Type 1	3 (23.1)	98 (24.3)	1.000
	Type 2	10 (76.9)	305 (75.7)	

### 3.2 Prevalence of diabetic retinopathy

**Figure 1:**
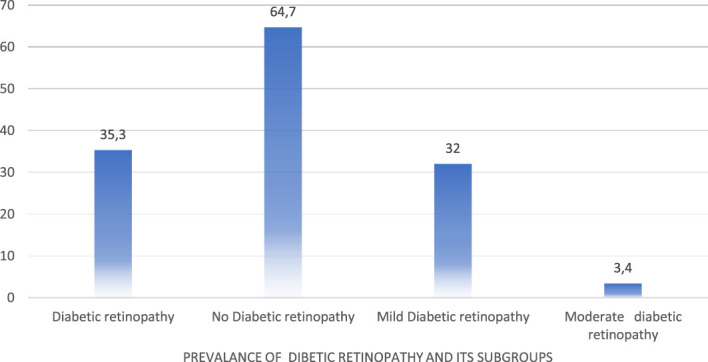
Prevalence of diabetic retinopathy and its subgroups.

The overall prevalence of DR among 416 patients was 35.4%, comprising 32% of mild non-proliferative DR (NPDR) and 3.4% of moderate NPDR (**Figure 1**).

In **Table 2**. Participants aged 55 and above had the highest proportion of diabetic retinopathy. The majority of participants in the aged group < 45-54 years had no diabetic retinopathy as compared to those with diabetic retinopathy. The proportion of participants with no matric certificates, and pensioners was higher in diabetic retinopathy as compared to non-diabetic retinopathy respectively (91.2 vs 81.4, *p=0.010*), (71.4 vs 9.7, *p=<0.001*). The proportion of unemployed, participants with matric certificates and alcohol consumption was higher in non-diabetic retinopathy as compared to diabetic retinopathy respectively (33.1 vs 19.7, *p=0.004*), (10.8 vs 3.4, *p=0.008*), (9.7 vs 7.1, *p= 0.001*).

**Table 2:** Comparison between socio-demographic profiles of participants with diabetic retinopathy and those without diabetic retinopathy.

Variables		Non-Diabetic retinopathy	Diabetic retinopathy	p-value
Male’s n (%)		67 (24.9)	34 (23.1)	0.721
Female’s n (%)		202 (75.1)	113 (76.9)	
Age	<45-54 years	89 (33.1)	23 (15.6)	<0.001
	≥ 55 years	180 (66.9)	124 (84.4)
Highest level of education	No matric certificate	219 (81.4)	134 (91.2)	0.010
	Matric certificate	29 (10.8)	5 (3.4)	0.008
	Post matric certificate	21 (7.8)	8 (5.4)	0.425
Employment status	Unemployed n (%)	89 (33.1)	29 (19.7)	0.004
	Employed n (%)	37 (13.8)	129 (8.2)	0.111
	Self-employed n (%)	6 (2.2)	1 (0.7)	0.429
	Pensioner n (%)	137 (50.9)	105 (71.4)	<0.001
Alcohol consumption n (%)		26 (9.7)	2 (7.1)	0.001
Smoking n (%)		2 (0.7)	3 (20.2)	0.351
Obesity n (%)		82 (30.5)	54 (36.7)	0.229

**Table 3**. Presents a comparison of the sociodemographic characteristics between participants with diabetes retinopathy and those without non-diabetic retinopathy. Type 2 DM participants with diabetic retinopathy had a higher proportion of pensioners, and participants with no matric as compared to those with non-diabetic retinopathy respectively (72.3 vs 51.1, *p=0.002*), (91.5 vs 81.7, *p=0.008*). Type 2 DM participants with non-diabetic retinopathy had a higher proportion of unemployment and alcohol consumption, and participants with matric as their highest level of education as compared to those with diabetic retinopathy respectively (33.2 vs 18.4, *p=0.002*), (9.2 vs 1.4, *p=0.002*), and (11.1 vs 3.3, *p=0.008*). In type 1 DM, there was no significant difference in the socio-demographic profiles between with participant’s diabetic retinopathy and those without diabetic retinopathy.

**Table 3:** Comparison of diabetic retinopathy and non-retinopathy with associated factors.

Variables		Type 1 Diabetic			Type 2 Diabetic		
		Non-Diabetic Retinopathy	Diabetic Retinopathy	P-value	Non-Diabetic Retinopathy	Diabetic Retinopathy	P-value
Age	<45-54 years	5 (71.4)	2 (33.3)	0.286	5 (71.4)	2 (33.3)	0.286
	≥ 55 years	2 (28.6)	4 (66.7)		2 (28.6)	4 (66.7)	
Highest level of education	No matric certificate	5 (71.4)	5 (83.3)	1.000	214 (81.7)	129 (91.5)	0.008
	Matric certificate	_	_	_	29 (11.1)	5 (3.5)	0.008
	Post matric certificate	2 (28.6)	1 (16.7)	1.000	19 (7.3)	7 (5.0)	0.524
Employment status	Unemployed	2 (28.6)	3 (50.0)	0.592	87 (33.2)	26 (18.4)	0.002
	Employed	2 (28.6)	0 (0.0)	0.462	35 (13.4)	12 (8.5)	0.193
	Self-employed	_	_	_	6 (2.3)	1 (0.7)	0.429
	Pensioner	3 (42.9)	3 (50.0)	1.000	134 (51.1)	102 (72.3)	<0.001
Alcohol consumption		2 (26.8)	0 (0.0)	0.462	24 (9.2)	2 (1.4)	0.002
smoking		0 (0.0)	1 (16.7)	0.462	2 (0.8)	2 (1.4)	0.614
Obesity		1 (14.3)	2 (33.3)	0.559	81 (69.1)	52 (36.1)	0.267

The predictors of DR are shown in **Table 4** after a logistic regression analysis model was used to assess the strength of a relationship between the dependent variable (i.e. DR) and independent factors. Old diabetic participants aged 55 and above, employed dietetic, and dietetic participants who were hypertensive were more likely to have diabetic retinopathy. DM participants with matric or more and DM participants who were alcohol consumers were less likely to have DR. Diabetic individuals who were smoking, hyperglycemic, poor glycemic control, and obese were associated with DR however, not statistically significant.

**Table 4:** Logistic regression between DR and associated factors.

Predictors		OR (95% CI)	p-value
Age	<45-54 years	Reference (1)	<0.001
	≥ 55 years	2.7 (1.6-4.4)	
Gender	Males	Reference (1)	0.686
	Females	1.1 (0.7-1.8)	
Educational level	< No matric	Reference (1)	0.038
	≥ Matric	0.6 (0.4-1.0)	
Employment status	Unemployed	Reference (1)	
	employed	1.4 (1.2-1.6)	<0.001
Alcohol drinking	Not drinking alcohol	Reference (1)	0.006
	Drinking alcohol	0.1 (0.03-0.6)	
Hyperglycemic state	Normal	Reference (1)	0.689
	Hyperglycemia (≥ 6.5 mmol/L)	1.1 (0.7-1.8)	
Glycemic control	Good glycemic control	Reference (1)	0.573
	Poor glycemic control	1.4 (0.38-5.6)	
Family history of DM	No family history	Reference (1)	0.621
	Family history	0.9 (0.6-1.4)	
Cigarette smoking	Not smoking cigarette	Reference (1)	0.266
	Smoking cigarette	2.8 (0.5-16.8)	
Systolic blood pressure	<130-139 mmHg	Reference (1)	0.006
	≥ 140 mmHg	1.4 (1.1-1.7)	
Body mass index (BMI)	<25.0-29.9 kg/m^2^	Reference (1)	0.233
	≥ 30 kg/m^2^	1.1 (1.0-1.3)	

## 4 Discussion

This study comprised type 1 and type 2 diabetes patients seeking treatment at public healthcare facilities in Maruleng, the local municipality of Mopani District, Limpopo Province. The overall proportion of diabetic retinopathy (DR) was 35.3%. DR in this study was more prevalent in females, type 2 DM patients, and older patients as in the other reported study in Spain (30) but contrary to other reported studies in Ethiopia (31), and Saudi Arabia (16), wherein the DR was more prevalent in males. However, only type 2 DM and patients over the age of 60 were consistent in these investigations. These findings are comparable to a systematic meta-analysis which reported a global prevalence of DR to be between 27% and 28.1% (25, 26), which was lower than the prevalence of our study, and this difference could be due to unequal healthcare systems around the world or year of publication (25).

The present study has shown a higher prevalence of DR than the prevalence of DR in the region of Europe, South East Asia, the Middle East, and North Africa, which was reported between 12.5% and 33.8% (32) contrary to a higher prevalence of DR in the region of Western Pacific (26). This could be due to the high prevalence of diabetes and other risk factors for non-communicable diseases in South Africa (33). Previous studies in South Africa carried out at the National Hospital in Bloemfontein (34), and Tshwane district (35) indicated the prevalence of DR to be 10.8% and 24.9%, respectively, which is lower than the proportion of DR in the current study. However, Mahomed et al. reported a higher prevalence of DR (71%) in their study which was carried out in KwaZulu-Natal at a provincial Hospital (28). This could be owing to the research methodology or kind of sample utilized to collect data, notably, the use of the diagnoses was categorized into 11 broad groups of eye conditions previous study (28), where there was a high possibility of participants over-reporting the prevalence. Thus the study elaborating the importance of using clinical confirmed diagnosis of DR

There was no relationship between DR and gender (both sexes) in this study. In accordance with the present Zhang et. (36), reported similar findings. However, Wat et al. reported DR to be prevalent in males (11). Similarly, several studies reported a positive association between the male gender and DR (37, 38). The inconsistencies between the present study and the study by Cui et al. (37), and Yin et al. (38), may be due to the difference in sample size and different geographical locations. In addition, the present study was conducted among participants aged 18 and above but the study by Cui et al, only considered participants aged 40 and above.

In the present study, participants aged 55 and above had the highest proportion of diabetic retinopathy as compared to those without it, and the participants aged <45-54 years had no diabetic retinopathy as compared to those with diabetic retinopathy. Binary logistic regression showed that participants aged 55 and above were 27 times more likely to have DR. In agreement with the present study Learned and Pieramici (2), reported old age to be positively associated with DR. Old age is reported to be associated with diabetes and hypertension (39). Diabetes and hypertension are reported to co-exist together which can ultimately lead to DR (11).

The proportion of unemployed participants was higher in non-diabetic retinopathy as compared to diabetic retinopathy. However, the proportion of pensioners was higher in diabetic retinopathy as compared to non-diabetic retinopathy. In the Logistic regression employed, participants were significantly associated with DR which concurs with the finding of the previous study reported by Thomas et al. (26). However, other studies reported different findings (31, 32).

The proportion of alcohol consumption was higher in non-diabetic retinopathy as compared to diabetic retinopathy. Logistic regression showed a negative association between alcohol consumption and DR. The findings of the present study concur with the study by Gupta et al. (40), who reported alcohol consumption to be negatively associated with DR (40). Similarly, Xu et al. (41) noted an association between baseline alcohol consumption and a lower prevalence of DR (41). However, other studies reported no association between alcohol consumption and DR (42, 43). The difference between the present study and other studies that reported the insignificant association between alcohol consumption and DR may be due to that in the present study there was no measurement of the alcohol quantity. The present study found no association between smoking and DR. These findings are similar to several studies that reported a non-significant association between smoking and DR (11, 44, 45).

A high systolic blood pressure (or hypertension) in the present study was significantly associated with DR, and this concurs with the findings of the other previously reported studies (12–16). The relationship between hypertension and diabetic retinopathy may be explained by the clinical observation that hypertension and diabetes usually coexist (11). Hypertension causes blood vessels in the eye to rupture and bleed, damaging the nerves in the eye, which ultimately results in the blockage of arteries and veins that carry blood to the retina and out of the retina (46, 47).

## 5 Conclusion

The prevalence of diabetic retinopathy among DM participants receiving chronic diabetic treatment from public or state-owned healthcare in Maruleng, Mopani district of Limpopo province in South Africa was 34%. DR was more prevalent in females, type 2 DM patients, and older DM patients. The age of DM patients, employment status, and high systolic blood pressure (hypertension) were significantly associated with an increased risk of developing DR.

## Data availability statement

The raw data supporting the conclusions of this article will be made available by the authors, without undue reservation.

## Ethics statement

The studies involving humans were approved by Turfloop Research Ethics Committee (TREC) (TREC/320/2017:PG). The studies were conducted in accordance with the local legislation and institutional requirements. The participants provided their written informed consent to participate in this study.

## Author contributions

DKM: Conceptualization, Methodology, Statistical analysis, Validation, Writing – review & editing. CBN: Conceptualization, Methodology, Statistical analysis, Validation, Writing - review & editing. RGM: Conceptualization, Validation, Writing - review & editing. KPS: Conceptualization, Validation, Writing - review & editing. EM: Conceptualization, Methodology, Writing - review & editing.
